# Workplace Bullying, Anxiety, and Job Performance: Choosing Between “Passive Resistance” or “Swallowing the Insult”?

**DOI:** 10.3389/fpsyg.2019.02953

**Published:** 2020-01-17

**Authors:** Mengyun Wu, Qi He, Muhammad Imran, Jingtao Fu

**Affiliations:** ^1^School of Finance and Economics, Jiangsu University, Zhenjiang, China; ^2^School of Management, Hainan University, Haikou, China

**Keywords:** workplace bullying, passive resistance, state anxiety, job performance, trait anxiety

## Abstract

Anxiety arising from workplace bullying is a key concern for job performance. Anxiety can explain the effects of workplace bullying: individuals may seek to deal with their anxiety by applying specific behaviors. However, anxiety research does not carefully distinguish between state anxiety and trait anxiety, and so the impact of anxiety in general has been seen as complex and contradictory. Individuals may respond to bullying and anxiety through “passive resistance” or by “swallowing the insult.” However, under what circumstances do individuals choose between these options? This paper summarizes the mechanisms of state anxiety and trait anxiety and uses cognitive balance theory to measure loss of self-control and the strategic choices. A moderated mediation model is presented for the relationship between workplace bullying and job performance using key variables of state anxiety and trait anxiety. Employee-supervisor pairs from 20 organizations and institutions from Tianjin, Jiangsu, and Hainan participated in a two-point longitudinal survey in 2019, 82.67% effective. Analysis verified that trait anxiety is the decisive perspective for choosing between “passive resistance” and “swallowing the insult.” This provides theoretical and practical contributions to psychology and organizational behavior research.

## Introduction

Workplace bullying is a persistent series of mistreatments of others in the workplace. It can include verbal criticism or direct personal attacks with the purpose of intentionally humiliating or belittling others (Adams and Bray, 1992). Workplace bullying leads individuals to doubt their concept of their own self and worth in the face of a dangerous environment (Attell et al., 2017), inducing psychological and physical discomfort or damage. As interpersonal conflict, workplace bullying represents a comprehensive behavior in the form of offense and insult. It is a negative interpersonal behavior formed on the basis of a formal or informal power imbalance (Ahmad, 2018). Any attempt at effective complaint or defense is likely to be met with silence or attack, resulting in serious adverse consequences for the victim’s mood.

Since its recognition as an issue in the workplace in the mid-1980s, workplace bullying has come to be seen as an increasingly serious example of workplace violence (Einarsen and Nielsen, 2015). Many employees are subjected to bullying of some kind at some point in their career. Chinese employees, however, are more likely to view their experience as typical or normal, given a culture of power orientation and obedience that is unique to Confucianism (Guo et al., 2015; Mengyun et al., 2018).

The negative consequences of workplace bullying may be viewed from different perspectives (Magee et al., 2017). First, employees may suffer from repeated negative behaviors from superiors, colleagues, or subordinates over a long period, causing psychological pressure and emotional damage. Such damage affects physical and mental health and an employee’s family life (Finstad et al., 2019). Second, workplace bullying has a strong negative outcome in victims’ low work efficiency and quality. This leads to considerable cost to an organization in a financial sense and also has a destructive effect on the organization’s growth.

Existing studies mostly draw on social psychology in analyzing the impacts of bullying on the victim. Among these is anxiety. Anxiety is the formation of complex emotional responses such as internal unrest and physiological discomfort (Laws and Bhatt, 2005). High levels of anxiety can impair individual task performance (Eysenck et al., 2007) and can explain the negative consequences of workplace bullying (Rodriguez-Munoz et al., 2015; Duru et al., 2018). Such views can integrate research on workplace bullying and the principles of ego depletion theory (which we address more fully below), in which an individual’s self-control resources largely promote or buffer the negative effects. Unavoidable occupational aggression in the organization occupies the limited self-control resources available to a victim and brings about more mental tension and psychological disturbance (Carvalho et al., 2018), inducing adverse consequences (Inzlicht and Kang, 2010). Workplace bullying is an exhausting experience that consumes physical and mental resources (Sprigg et al., 2019). It eventually leads to severe exhaustion of self-control resources and failure of that self-control.

However, some scholars also believe that anxiety can motivate individuals to avoid failure (Eysenck, 2010). Most studies have identified negative emotional experiences as mediating variables in studying effects of workplace bullying (Namie, 2014). For example, anxiety is argued to have an adaptive value as a crisis warning of psychological barriers, it drives cognitive processing, and it considers taking risk avoidance measures as early as possible. Individuals may then devote more attention to optimizing coping strategies so as to ensure task completion (Moriya, 2018). Such findings reflect the complex cognition of the relationship between emotions and subsequent behavior, and they prompt more questions. Can anxiety motivate employees to have negative workplace behaviors? Can the theory of ego depletion fully explain the effect of workplace bullying on employee performance?

The mixed findings about the impact of anxiety have mainly arisen from a failure to distinguish the dimensions of anxiety. State anxiety is anxiety about a situation, whereas trait anxiety is the individual’s anxiety level as a personal characteristic. (We discuss these further below.) Failure to separate these dimensions has led most scholars to focus only on the emotional responses to stressors and to ignore cognitive processing. This paper therefore proposes a more detailed theoretical view on the behavioral response of individuals to the process of self-control resource consumption in workplace bullying. It argues that trait anxiety can moderate the individual’s anxiety perception through influencing the cognitive evaluation system. Trait anxiety represents a personality trait. Individuals can maintain a habitual anxiety mood during different periods and situations, identifying threats or dangers in their external environment (Spielberger, 1985). Individuals with high trait anxiety may not respond to workplace bullying through irrational decision making. They are more likely, instead, to choose silence and endurance, and to prefer negative withdrawal strategies that tolerate and ignore bullying. This is more of a preconceived behavioral motivation (Moriya, 2018).

**FIGURE 1:**
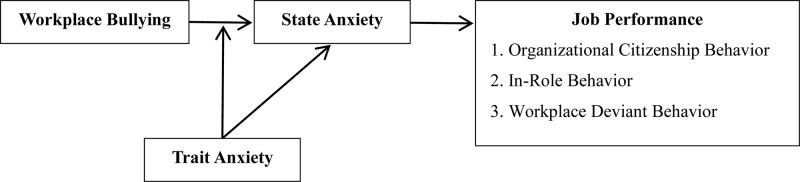
Theoretical model.

The moderated mediation model presented in Figure 1 relates workplace bullying and job performance through key variables of state anxiety and trait anxiety. It can be used to explain when an individual displays “passive resistance” and when he or she chooses to “swallow the insult,” or, in a similar metaphor, “turn a blind eye” to the behavior inflicted. This in turn can also explain under what conditions workplace bullying will have a negative impact on the individual’s performance. It provides a theoretical explanation for the conflicting findings in previous studies.

This theoretical model provides an innovative contribution.

First, it focuses on the mediating role of state anxiety through a literature review on hostile interpersonal treatment. It summarizes how workplace bullying leads to counterproductive work behavior of employees through ego depletion. Then, focusing on the moderating effect of trait anxiety, this paper proposes a more accurate theoretical framework than does the existing mediating model to investigate whether workplace bullying is related to employees’ negative behaviors in the workplace. The theoretical model therefore reveals how state anxiety and trait anxiety together contribute to the relationship between workplace bullying and employees’ negative workplace behavior.

Second, the theoretical model can explain contradictory findings about the relationship between workplace bullying and job performance. Theoretical findings on the relationship between workplace bullying and organizational citizenship behavior are contested. Some studies show a negative correlation (Magee et al., 2017), in that employees may retaliate against the organization openly through behaviors that are harmful to the organization’s interests, or behaving more covertly, they seek to calm their emotional fluctuations. However, other studies have also shown no significant correlation between the two (Liu, 2016). In the moderated mediating-effect model of this paper, we show that state anxiety under a condition of high trait anxiety cannot effectively exert a mediating effect. Our paper thus improves general understanding of individual behavior choice and deals with the weakness of previous studies.

Third, the construction of a theoretical model to describe the boundary conditions of individual choice between “passive resistance” and “swallowing the insult” contributes to the theory of cognitive balance. When trait anxiety is low, bullied individuals may develop behaviors that are consistent with state anxiety. However, in a passive position, where power is weak and resources are scarce, they do not resort to actions that aggravate conflicts but choose indirect passive coping strategies. Individuals with high trait anxiety, we found, are able to avoid the threat of negative stimuli based on their stable anxiety experiences and are more likely to choose avoidance via inaction in order to respond to emotional abuse. This is not out of loyalty to the organization, nor is it a coping strategy (Reknes et al., 2016; Birkeland et al., 2017). It is a complex expression of a tendency to acquiesce. In the field of organizational behavior research, cognitive balance theory, as an internal perspective of individual information processing, can explain such “unexpected” and “expected” cognitive situations. Hence, through integration of ego depletion theory and cognitive balance theory, this paper investigates the choice between “passive resistance” and “swallowing the insult” by adopting trait anxiety as a moderating variable. This deepens the research on the relationship between workplace bullying and employee job performance.

## Theoretical Basis and Hypothesis

### Ego Depletion, Workplace Bullying, and State Anxiety

Emotional assessment theory, which explains the formation process of emotional responses, states that the negative emotions of individuals in an organization are derived from the information that they perceive after observing and judging the surrounding environment and environmental changes that may hinder the realization of their own goals or interests (Scherer et al., 2001). State anxiety is an immediate and volatile negative emotion that occurs spontaneously in the face of a specific moment, event, or challenge (Endler and Kocovski, 2001). As a relatively transient psychological condition, it involves cognitive arousal and physiological activation.

According to the theory of ego depletion, state anxiety is the psychological and physiological reaction to differing degrees of mental distress after the individual receives the stress source and forms a pressure perception. This is accompanied by continuous self-control resource consumption, which induces excessive psychological pressure, mental frustration, and helplessness (Kakarika et al., 2017; Carvalho et al., 2018). The relationship between poor work environment and low mood state is therefore rooted in the theoretical framework of ego depletion theory (Li and Zhang, 2017).

Baumeister et al. (1998) proposed the theory of ego depletion to explain workplace bullying effects. Ego depletion theory argues that such bullying can instigate an individual’s attitudes and behaviors consistent with negative psychological burdens in the face of emotional responses that are situational and temporary (Hofmann et al., 2009).

Ego depletion is based on negative psychological self-control ability. The lack of input energy means that the bullying victim cannot ensure that subsequent behavior is consistent with the self-control goal. Studies have shown that state anxiety accompanied by impaired cognitive control is an important factor in inducing employee bad behavior (Li and Wu, 2016). Employee depression further affects follow-up work motivation and resource investment in work roles and task management (Wang, 2017). Ego depletion explains how state anxiety can negatively affect job performance: individuals may assess the workplace as of low quality, or a place with more negative than positive interpersonal relationships, and they may have unequal internal relations with others, resulting in a perfunctory work attitude (Du et al., 2017).

In cases of workplace bullying, ego depletion means that individuals need to disperse their attention resources and psychological energy in order to suppress habitual thinking tendencies and impulsive behavioral responses (Dewall et al., 2008), which are considered the root causes of subsequent self-control failure and self-management dilemmas. Individuals process information related to stress sources according to their own cognitive state, and they generate perceptions of hostility to the organizational atmosphere and the implications of poor interpersonal relations (Du et al., 2017). Poor interpersonal communication in the organizational environment therefore leads to the formation of uncertain and unsafe evaluation and judgment in employees, and this then negatively influences their emotional state by way of ego depletion (Pan et al., 2017).

The individual forms negative emotions in a stressful situation through the process of ego depletion, and this in turn endangers task performance. Individuals with high state anxiety reduce their cognitive range based on their own low emotional stability, and so are more likely to present a lack of organization role performance and to avoid the formation of organizational citizenship behavior. This may seriously damage in-role behavior, present a workplace deviant behavior of immoral or anti-normative action (Mawritz et al., 2017), and feed back into a low safety climate for the organization or result in upgrading interpersonal conflict. While low ego depletion can maintain interpersonal relationships and standardized production behavior motivation (Zhang and Zhang, 2017), individuals may try to maintain a normalized performance level with less adaptive avoidance due to their extreme emotions.

### Workplace Bullying, Trait Anxiety, and Cognitive Balance

According to the theory discussed above, an increase of state anxiety may result in non-direct, counterproductive work behaviors, such as work slowdown, reduced productivity, and reduced performance. However, scholars have not distinguished the dimensions of anxiety. In the absence of this distinction, the effects of anxiety on performance may be highly variable. Job performance has, broadly, three dimensions: organizational citizenship behavior, in-role behavior, and workplace deviant behavior (Hoel et al., 2001). Although empirical studies link increases in the level of anxiety and decline of organizational citizenship behavior, in-role behavior, and growth of workplace abnormal behavior (Mawritz et al., 2017), other theoretical and empirical research conflicts with these conclusions, arguing that correlation between anxiety level and performance results is not proved (Eysenck, 2010). The relationship between workplace bullying and performance results is similarly disputed (Liu, 2016; Magee et al., 2017). In view of these differences, this study argues that the key factors of individual ego depletion not only depend on stress intensity but also involve the individual’s acquired personality and coping style.

Spielberger (1985) described trait anxiety as a relatively permanent personality characteristic. So individuals can perceive a wide range of external stimulation with a fixed emotional response and frequency, and show a consistent response in various environments. Trait anxiety represents a kind of long-term emotional experience, whose level depends on the individual’s personality characteristics formed in the socialization process. Individuals have relatively complete and unique potential emotional tendencies toward external stimulus events, and they are not easily influenced by stress factors to initiate emotional feelings. They can thus maintain relatively stable sensory and cognitive feedback in different periods or situations. High trait anxiety individuals show unique recognition, processing orientation, and obstacle removal for hazardous information, and maintain a high level of vigilance for realistic clues to these (Graham and Shin, 2018). They thus show emotional consistency and make conservative decisions when processing external information.

Trait anxiety is a construct distinct from state anxiety. The level of state anxiety depends on the intensity of the stressor – the event – and individual sensitivity, which can be measured by external stimuli or fluctuating emotions of relatively short duration (Spielberger et al., 1983). However, trait anxiety is a stable personality attribute, which can be distinguished by its consistent emotional characteristics. It is not related to the level of state anxiety. Individuals may generally understand external work events and internal psychological experience as a danger signal in daily life, and gradually form a locking pattern related to a risk’s cognitive content. This is accompanied by psychological discomfort such as sensitivity and worry. The cognitive style and structural components of trait anxiety can distinguish under what conditions the individual’s anxiety consciousness will present as a sustained and stable mechanism that is only weakly related to the stress situation and the nervous implication of the stimulus event. Considering that the measurement of trait anxiety is directly determined by the individual characteristics and psychological structure of the anxiety disorder that will directly affect the development and channeling of emotions under the pressure source, it is more suitable for use in theoretical research to test whether trait anxiety can play an important role in the moderation of stress-related state anxiety.

Cognitive balance represents the coexistence without pressure of individuals’ self-perception and emotional experience in objective situations. In this study, the premise of cognitive balance is that the external stimuli perceived by an individual in a stressful situation can be consistent with the individual’s conventional thinking mode, so that the individual’s inner mind can operate rationally. As a special source of anxiety experience in the anxiety cognitive system, trait anxiety can be considered a benchmark for individuals in seeking cognitive balance in a context of stress (Zhu, 1989). Individuals with high trait anxiety rely on their original cognitive experience to associate reasoning and stimulate the cognitive balance system by matching external threat information. The balance between the work cognitive state and self-emotional awareness makes emotional detection more automatic. Under such circumstances, individuals can carry out effective emotional control and processing (Moriya, 2018), without causing themselves extreme emotional fluctuations and negative emotional disclosure, and avoiding the serious consequences caused by cognitive dissonance (Zhao et al., 2016). Individuals with high trait anxiety therefore agree with their self-consciousness through feedback of cognitive content, which buffers the negative effect of negative information on emotions in a stressful situation and alleviates the loss process of self-control resources. The cognitive habits of individuals with low trait anxiety are far below the stress line, and the unfair treatment they suffer is incompatible with their cognitive structure, thus leading to formation of a kind of emotional labor, which has more obvious hindering effects on emotional relief.

Individual differences play a key moderating role in determining emotional experience and behavioral response after bullying. Not all people show adverse consequences for performance after being bullied. Trait anxiety, as an important influencing factor of individual emotional stability and behavior change under stress, can adjust the effect of workplace bullying on individual behavior performance through state anxiety. According to cognitive balance theory, individuals evaluate workplace bullying events based on their own trait anxiety, activate their cognitive evaluation system consistent with trait anxiety, and respond to perceived negative balance with tacit psychology and compliant behavior after seeking for an inner match (Lee et al., 2018).

The anxiety experience of high trait anxiety individuals is a persistent psychological cognition. Bullying events in organizational situations can be consistent with their stable anxiety experience. They alleviate their sensitive response to negative information based on internal pressure, reduce state anxiety by reducing ego depletion, and reduce the negative effect on behavioral performance, so that they are more persistent with their goals and tasks (Moriya, 2018). Individuals with low trait anxiety have lower immune limits to stress stimulation, and most identify the external environment as having an objective existence of low risk. However, the cognitive difference between the meaning of hostile attack through workplace bullying and the original psychological pattern induces intense discomfort, and subsequent ego depletion strengthens the individual’s inner sense of situational anxiety, undermines behavioral motivation (Magee et al., 2017), and involves deteriorating performance.

### Summary

Feng (2016) reports that workplace bullying leads to increased state anxiety. This finding agrees with the main argument of ego depletion theory, which states that such bullying can generate attitudes and behaviors consistent with negative psychological burdens in the face of situational and temporary emotional responses (Hofmann et al., 2009). The passive resistance that results reduces organizational citizenship behavior and in-role behavior, and increases workplace deviant behavior (Mawritz et al., 2017). State anxiety can mediate the negative impact of workplace bullying on job performance, but the anxiety experience at this point does not have personality characteristics. Cognitive processing depends on an individual’s stable trait level (Zhu, 1989). High trait anxiety individuals’ accurate prediction of stress situations improves their stress resistance and tolerance to bullying events, and reduces self-control resource loss and situational anxiety experience (Moriya, 2018): individuals seek to “swallow the insult” and strive to maintain normal behavior performance (Lee et al., 2018). This theoretical framework thus explains the relationship between workplace bullying and behavior and when those with trait anxiety choose “passive resistance” and when they choose to “swallowing the insult.” This decision can be integrated into a moderated, mediating model to explain the effect of workplace bullying on job performance through state anxiety, such that the mediating effect of state anxiety under the influence of different levels of trait anxiety may be stronger or weaker (Edwards and Lambert, 2007).

Based on this discussion, this paper proposes the following hypotheses:

***Hypothesis 1:*** Workplace bullying has a positive effect on state anxiety.

***Hypothesis 2:*** Trait anxiety negatively moderates the relationship between workplace bullying and state anxiety.

***Hypothesis 3:*** Trait anxiety negatively moderates the mediating effect of the relationship between workplace bullying and organizational citizenship behavior, in-role behavior, and workplace deviant behavior.

## Research Method

### Investigation Procedures

In this study, twenty organizations and institutions in Tianjin, Jiangsu, and Hainan were investigated through online questionnaires. Organization type included public institutions, state-owned enterprises, joint ventures, and private enterprises. Prior to the survey, we contacted relevant human resource department managers and secured their consent and help, then issued questionnaires based on the email addresses they provided. The investigation required the subjects to participate with their direct supervisor. The specific procedures were described to subjects prior to commencement. We also informed them that data collected were only to be used for academic research. Hence, this research manipulated the process in terms of normative question-and-answer procedures and anonymous answers. Participants were asked questions related to their own demographic information, workplace bullying, and trait anxiety, and then their level of state anxiety was assessed a month later. At this latter time, the direct supervisor also needed to evaluate the organizational citizenship behavior, in-role behavior, and workplace deviation behavior of the subjects.

Because the study involved human participants, it was reviewed and approved by the ethics committee in the School of Finance and Economics of Jiangsu University, Zhenjiang, China. Written informed consent was not required, in accordance with national legislation and institutional requirements. Consent was inferred through completion of the survey.

We issued 300 questionnaires. Fifty-two pairs of samples from seven organizations were excluded due to refusal to participate, withdrawal, or invalid completion of the questionnaire, leaving 248 pairs with complete data (an effective response rate of 82.67%). The proportion of males was 57.66% (SD = 0.50), the average age was 33.61 years (SD = 7.44), and the average working experience was 12.76 years (SD = 8.21). Respondents held different grades of position: junior, 28.63%; intermediate, 44.35%; assistant senior, 14.52%; and senior, 12.50%.

### Variable Measurement

All English scales were translated and back-translated, following Brislin (1980). Questionnaires were scored with a five-level Likert evaluation.

#### Workplace Bullying

The negative behavior questionnaire compiled by Einarsen et al. (2009), revised by Jiang et al. (2011), requires subjects to answer four questions. An example item is “someone is telling tales, making or spreading rumors about you.” In this study, the Cronbach’s alpha reliability coefficient was 0.797.

#### State Anxiety

Using the state-trait anxiety questionnaire revised by Spielberger et al. (1983), four items in the state anxiety sub-table were extracted, and the subjects were asked to conduct self-evaluation. An example item is “you feel calm.” Cronbach’s alpha was 0.735.

#### Job Performance

The 21 scales of Williams and Anderson (2016) were selected to evaluate the organizational citizenship behavior and in-role behavior of the subjects. Of these, two items were selected to measure interpersonal organizational citizenship behavior, two to measure organizational citizenship behavior, and four to measure in-role behavior. Four items in Bennett and Robinson (2000) scale were selected to measure interpersonal-oriented deviation and organizational-oriented deviation, respectively. The subject’s direct supervisor evaluated the subject’s behavior. Example items were “you assist colleagues,” “you can complete job duties,” and “you will use authority to seek personal benefits.” Cronbach’s alpha coefficients were 0.687, 0.699, and 0.829, respectively.

#### Trait Anxiety

The revised State-Trait Anxiety Scale (Spielberger et al., 1983) of Li and Qian (1995) was used to measure the anxiety tendency of the subjects’ personality traits. Two positive items and two negative items were selected. An example item is “you hope to be as happy as others.” Cronbach’s alpha was 0.817.

### Data Analysis

Edwards and Lambert (2007) show that the moderated mediator hypothesis can be verified by checking whether the moderating effect exists and whether the mediating effect changes correspondingly because of different levels of the moderating variable. In this study, two multiple regression models were used to test the moderated mediating hypothesis (Ng et al., 2008): first, to examine whether workplace bullying affects state anxiety, and second, whether standardized treatment of workplace bullying and trait anxiety eliminates multicollinearity, and then to examine the effect of its interaction items on state anxiety.

By integrating the regression model, the direct effect of workplace bullying on state anxiety and the mediating effect of state anxiety at different trait anxiety levels can be obtained. Then, through the bootstrap method, the significance test of the direct effect and indirect effect, and the difference value of the regression model were conducted.

### Reliability Test and Validity Test

Exploratory factor analysis results showed that the KMO value was 0.875, the chi-square from Bartlett’s spherical test value was 2895.106, the significance level was less than 0.001, and the data matrix was correlated. In the absence of rotation, six factors with characteristic value greater than 1 were generated; the accumulated interpretation of variance variation was 65.015%. The variance variation degree of the first factor was 34.938%, which was 40% lower than the empirical standard value. Six principal components are extracted from the factor loading matrix, which is consistent with the number of variables set in this paper, and the absolute value of factor loading for each item is higher than the recommended standard of 0.5.

Since the main variables used in this study include multidimensional variables, confirmatory factor analysis is required to test their validity. We used the AMOS software package to perform the confirmatory factor analysis. The results are shown in Table 1: data-fitting indicators of workplace bullying, state anxiety, job performance, and trait anxiety all reach a reasonable range, and each variable has good aggregation validity.

**TABLE 1:** Confirmatory factor analysis.

**Variable**	**χ^2^**	**df**	**χ^2^/df**	**RMSEA**	**CFI**	**IFI**	**RMR**	**TLI**
Workplace bullying	6.996	2	3.498	0.101	0.985	0.985	0.031	0.955
State anxiety	3.953	2	1.976	0.063	0.992	0.992	0.026	0.977
Job performance	156.735	27	3.073	0.092	0.904	0.905	0.049	0.876
Trait anxiety	3.132	2	1.566	0.048	0.997	0.997	0.012	0.990

*RMSEA, root mean square error of approximation; CFI, comparative fit index; IFI, incremental fit index; RMR, root mean square residual; and TLI, Tucker Lewis Index.*

### Descriptive Statistics and Correlation Analysis

The mean value, SD, Cronbach’s alpha, and correlation coefficient of the variables are shown in Table 2: workplace bullying is significantly correlated with state anxiety (*r* = 0.58, *p* < 0.001); status anxiety is significantly correlated with organizational citizenship behavior (*r* = −0.36, *p* < 0.001), in-role behavior (*r* = −0.56, *p* < 0.001), and workplace deviance behavior (*r* = 0.059, *p* < 0.001); trait anxiety is significantly correlated with state anxiety (*r* = −0.32, *p* < 0.001); and trait anxiety is significantly associated with organizational citizenship behavior (*r* = 0.38, *p* < 0.001), in-role behavior (*r* = 0.39, *p* < 0.001), and workplace deviance behavior (*r* = −0.47, *p* < 0.001). These results preliminarily support the subsequent regression analysis.

**TABLE 2:** Descriptive statistics and correlation analysis.

	**Mean**	**SD**	**1**	**2**	**3**	**4**	**5**	**6**	**7**	**8**	**9**	**10**	**11**
1. Gender	0.42	0.50	−										
2. Age	33.61	7.44	0.25^∗∗∗^	−									
3. Working experience	12.76	8.21	0.22^∗∗^	0.89^∗∗∗^	−								
4. Position	2.11	0.96	0.28^∗∗∗^	0.16^∗^	0.22^∗∗∗^	−							
5. Organization	2.79	0.96	–0.06	–0.11	−0.16^∗^	0.24^∗∗∗^	−						
6. Workplace bullying	3.66	0.89	–0.06	0.01	0.01	−0.13^∗^	–0.25^∗∗∗^	0.797					
7. State anxiety	3.62	0.77	0.12	–0.09	–0.08	–0.10	–0.32^∗∗∗^	0.58^∗∗∗^	0.735				
8. Organizational citizenship behavior	2.37	0.71	−0.13^∗^	–0.05	–0.03	0.02	0.01	–0.45^∗∗∗^	–0.36^∗∗∗^	0.687			
9. In-role behavior	2.57	0.59	0.01	–0.06	–0.05	0.06	0.10	–0.47^∗∗∗^	–0.56^∗∗∗^	0.39^∗∗∗^	0.699		
10. Workplace deviant behavior	3.47	0.83	0.02	0.02	0.01	0.01	−0.14^∗^	0.55^∗∗∗^	0.059^∗∗∗^	–0.47^∗∗∗^	–0.60^∗∗∗^	0.829	
11. Trait anxiety	3.07	0.69	–0.05	0.24^∗∗∗^	0.21^∗∗^	–0.02	–0.12	–0.27^∗∗∗^	–0.32^∗∗∗^	0.38^∗∗∗^	0.39^∗∗∗^	–0.47^∗∗∗^	0.817

*Note: ^∗^*p* < 0.05, ^∗∗^*p* < 0.01, ^∗∗∗^*p* < 0.001.*

### Moderated Mediating Effects

We use hierarchical linear regression to test our research hypotheses. As shown in Table 3, workplace bullying has a significant positive impact on state anxiety (β = 0.470, *p* < 0.001). Hypothesis 1 is supported. The interaction items of workplace bullying and trait anxiety have a significant negative impact on state anxiety (β = −0.168, *p* < 0.001). This is different from the direction of state anxiety affected by workplace bullying and can explain the 4.40% of variation of state anxiety. This supports Hypothesis 2.

**TABLE 3:** Moderating effect of trait anxiety.

	**State anxiety**			
	**Model 1**	**Model 2**	**Model 3**	**Model 4**
Gender	0.217^∗^	0.266^∗∗^	0.223^∗∗^	0.192^∗^
Age	–0.006	–0.008	–0.002	0.001
Working experience	–0.010	–0.008	–0.010	–0.010
Position	–0.021	0.003	0.002	–0.009
Organization	–0.260^∗∗∗^	–0.153^∗∗^	–0.182^∗∗∗^	–0.179^∗∗∗^
Workplace bullying		0.470^∗∗∗^	0.417^∗∗∗^	0.312^∗∗∗^
Trait anxiety			–0.212^∗∗∗^	–0.169^∗∗^
Workplace bullying × trait anxiety				–0.168^∗∗∗^
R square	0.136	0.411	0.441	0.484
Adjust R square	0.118	0.396	0.424	0.467
R square changes	0.136	0.275	0.029	0.044
F	7.629^∗∗∗^	28.047^∗∗∗^	27.004^∗∗∗^	28.052^∗∗∗^

*Note: ^∗^*p* < 0.05, ^∗∗^*p* < 0.01, ^∗∗∗^*p* < 0.001.*

Subsequently, in order to test the moderated mediating effects, the effect values under different trait anxiety levels were calculated by the bootstrap method (Ng et al., 2008). Analysis of the data shows that the strength of the relationship between workplace bullying and state anxiety depends on the individual’s trait anxiety level. Under the condition of low trait anxiety, workplace bullying has a stronger predictive power for state anxiety (*P* = 0.599, *p* < 0.001), and its difference is significant ([0.321] – [0.599] = −0.277, *p* < 0.01).

**FIGURE 2:**
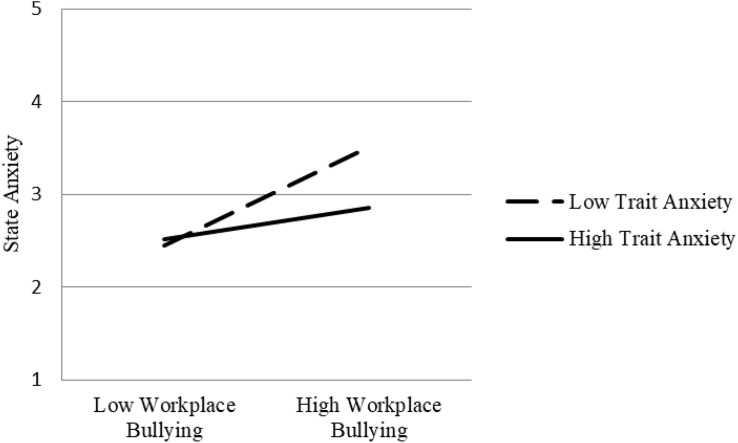
Schematic diagram of interaction effect (state anxiety).

Figure 2 shows the direction and trend of the relationship between workplace bullying and state anxiety at different trait anxiety levels. The positive correlation between these was stronger and higher at lower levels of trait anxiety and is expressed graphically in Figure 2.

**FIGURE 3:**
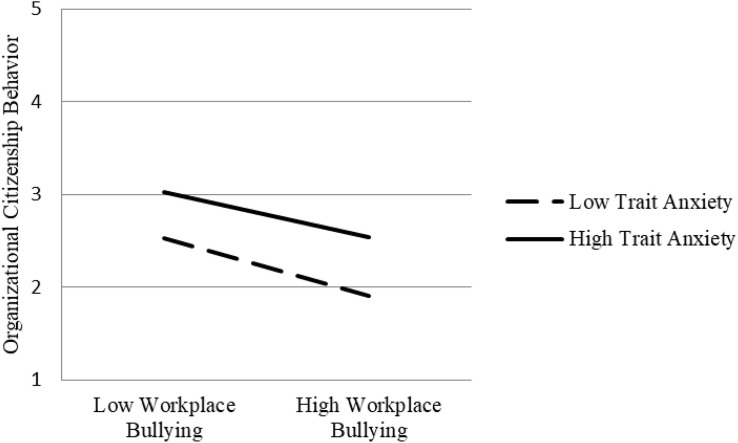
Schematic diagram of interaction effect (organizational citizenship behavior).

**FIGURE 4:**
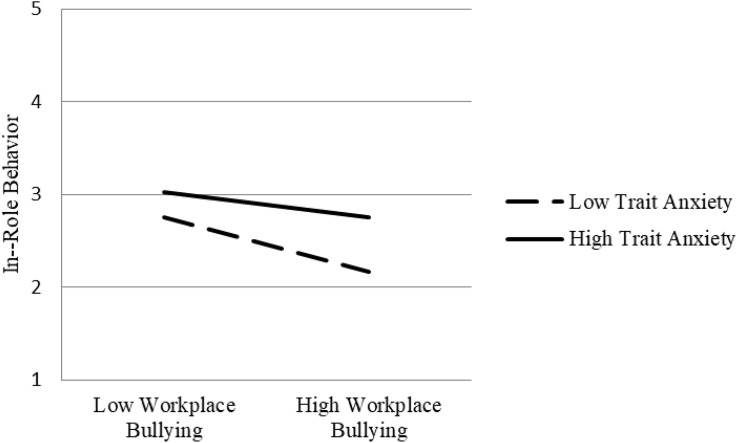
Schematic diagram of interaction effect (in-role behavior).

**FIGURE 5:**
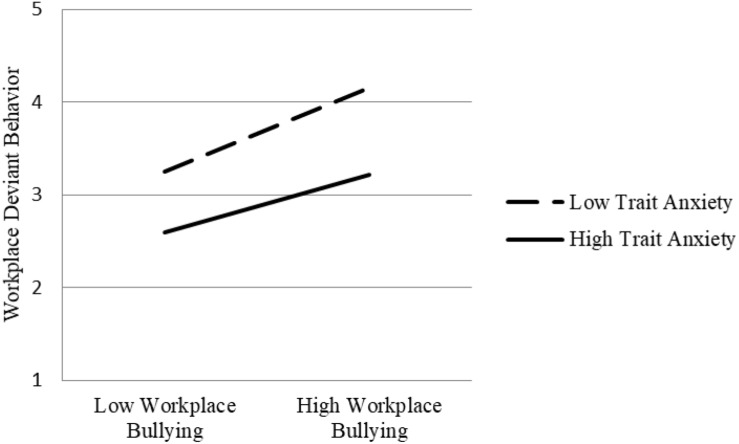
Schematic diagram of interaction effect (workplace deviant behavior).

In view of the moderating effect of trait anxiety of workplace bullying on status anxiety, it is necessary to test whether the indirect effect of workplace bullying on job performance also depends on trait anxiety level. Table 4 shows that, under the condition of low trait anxiety, the indirect effect of workplace bullying on organizational citizenship behavior (*P* = 0.032, *p* < 0.01), in-role behavior (*P* = 0.150, *p* < 0.001), and workplace deviant behavior (*P* = 0.213, *p* < 0.001) is stronger, and the indirect effect intensity is significantly dependent on trait anxiety level ([0.026] – [0.032] = 0.006, *p* < 0.05; [−0.094] – [−0.150] = 0.056, *p* < 0.05; [0.117] – [0.213] = −0.096, *p* < 0.05). Figures 3–5, respectively, show the indirect effects of workplace bullying on organizational citizenship behavior, in-role behavior, and workplace deviant behavior under different trait anxiety conditions. When the trait anxiety level is low, the indirect effect is stronger. The results support Hypothesis 3.

**TABLE 4:** Moderated mediating effects.

	**Trait anxiety**	**Direct effect**	**Stage 1**	**Stage 2**	**Indirect effect**	**Total effect**
Organizational citizenship behavior	High	–0.064	0.321^∗∗∗^	–0.081	–0.026^∗∗^	–0.090
	Low	–0.440^∗∗∗^	0.599^∗∗∗^	–0.053	–0.032^∗∗^	–0.471^∗∗∗^
	Difference	0.376^∗∗∗^	–0.277^∗∗^	–0.028	0.006^∗^	0.381^∗∗∗^
In-role behavior	High	0.000	0.321^∗∗∗^	–0.294^∗∗∗^	–0.094^∗∗∗^	–0.094
	Low	–0.241^∗∗∗^	0.599^∗∗∗^	–0.251^∗∗∗^	–0.150^∗∗∗^	–0.391^∗∗∗^
	Difference	0.241^∗∗∗^	–0.277^∗∗^	–0.043	0.056^∗^	0.297^∗∗∗^
Workplace deviant behavior	High	0.021	0.321^∗∗∗^	0.363^∗∗∗^	0.117^∗∗∗^	0.138
	Low	0.494^∗∗∗^	0.599^∗∗∗^	0.355^∗∗∗^	0.213^∗∗∗^	0.706^∗∗∗^
	Difference	–0.473^∗∗∗^	–0.277^∗∗^	0.007	−0.096^∗^	–0.569^∗∗∗^

*Note: ^∗^*p* < 0.05, ^∗∗^*p* < 0.01, ^∗∗∗^*p* < 0.001.*

## Discussion, Implications, and Limitations

This study reviewed the results of workplace bullying and its negative influence, and the theoretical basis for staff in choosing between “passive resistance” or “swallowing the insult” when faced with workplace bullying. The study posited trait anxiety and state anxiety as mediating variables of workplace bullying effects, and proposed a moderated mediation model in order to better understand the mechanism of workplace bullying effects on job performance. The results of the data analysis reveal the mediating mechanism and moderating mechanism of workplace bullying effects. Hence, the hypothesized model constructed in this study not only can enhance the theoretical relationships between workplace bullying, anxiety, ego depletion theory, and the theory of cognitive balance, but can also provide a theoretical basis for promoting changes in practice.

### Theoretical Implications

The theoretical framework of this paper explains the impact of workplace bullying on job performance. It can thus explain how negative interpersonal behavior affects job performance in general situations. Current studies on workplace bullying are based more on the ego depletion process and see anxiety as an outcome variable, while negative interpersonal behaviors generally involve interpersonal disputes or work-related friction and conflict (Du et al., 2017). Anxiety is therefore an inevitable result of negative interpersonal treatment. This paper describes the potential explanatory power of the theoretical model and provides a common framework for understanding how negative interpersonal behavior affects organizational results. The research findings on negative interpersonal behavior are complex, and hence, this paper integrates them into a “threat of ego depletion” model through the common points in the structure.

The findings in this paper also enrich the literature on anxiety. The theoretical findings about choosing between “passive resistance” and living with adverse behavior (“swallowing the insult”) have been a controversial topic in the field of organizational behavior. However, our study points out that cognitive balance is an important motivation for individuals to maintain their job performance. This indicates that the “passive resistance” proposition derived from the theory of ego depletion may be flawed. This paper argues that such confusion results from the failure to distinguish state anxiety and trait anxiety in the model. When the anxiety held by an individual is in fact clinical anxiety, it feeds back into the ego depletion process by reducing work investment. When the anxiety held by individuals is personality anxiety, individuals do not respond to stress situations by adjusting their organizational behavior, but maintain their previous behavior pattern. Therefore, based on the ego depletion theory and the cognitive balance theory, this paper proposes a boundary model to describe the influences on the individual’s behavioral motivation. The individual’s choice of strategies – in effect, resistance or acceptance (our “passive resistance” or “swallowing the insult”) – should be referred to the moderating effect of the individual’s trait anxiety. Follow-up research on this choice can continue to take trait anxiety into account and build a more scientific and rational model.

The conclusions of this paper can also supplement the weak links in study results related to trait anxiety. In general, studies on trait anxiety pay more attention to possible adverse effects: the negative feedback related to trait anxiety gradually becomes fragile physical and mental harm (Wei, 2016), causing more serious behavioral disorders. However, this paper points out the more positive side of trait anxiety: individuals can avoid excessive ego depletion in accordance with the cognitive balance of the stress situation and develop psychological peace within themselves. Combined with previous research findings, we can conclude that the moderating effect of trait anxiety may differ according to differing outcome variables: although high trait anxiety can buffer economic or social pressures in the short term, it enables employees to continue to work and meet organizational performance standards. However, surface control effects based on emotional reasoning are likely to be achieved at the expense of individual long-term sacrifice (Yu et al., 2016), and they deteriorate in the context of economic crisis (Mucci et al., 2016; Ketilsdottir et al., 2019). Subsequent studies can integrate organizational and individual results into the same model for further validation.

### Practical Implications

Conceptualizing the impact of workplace bullying on job performance through the mediated model can also provide a reference for organizational practices. First, by understanding the mediating mechanism of the impact of workplace bullying, we can better understand how workplace bullying affects job performance. Given that state anxiety has been shown to be associated with stress situations, negative information and events are likely to elicit individual anxiety responses (Kakarika et al., 2017). Hence, the organization can eliminate state anxiety caused by potential organizational problems through action and policy and thereby reduce the negative impact on work investment. Strategies might include, for example, implementing anti-bullying policies and severely sanctioning bullies, encouraging diversity and inclusiveness in the workplace, and creating a good organizational climate to improve employee psychological adaptability, ensuring healthy working hours, giving employees appropriate work autonomy and incentives, reducing self-control resource consumption, and guiding employees to keep their goals in focus.

Second, by outlining the moderating mechanisms that work on workplace bullying, we can better understand the boundary conditions under which workplace bullying affects job performance. In the context of workplace bullying, individuals with low trait anxiety experience a dramatic reduction in job performance. As a result, organizations may want to select employees with higher trait anxiety. However, high trait anxiety can also cause serious damage to individuals and the whole organization, such as damage to the mental state and physical health of employees (Wei, 2016). In view of the insufficiency of current research results, more careful discussion is still needed before making specific recommendations to organizational managers in this regard.

### Research Limitations and Future Directions

Limitations should also be considered. Are our data analysis results representative (Highhouse and Gillespie, 2009)? The research and sample collecting method in this paper is single, and cultural differences and expression habits also affect employee understanding of the content of the scale. Thus subjectivity and homology cannot be avoided, and the data obtained cannot accurately test the hypotheses. For example, while this article concludes that trait anxiety can mitigate the effects of workplace bullying on state anxiety, the implications of trait anxiety also agree with other views. On the one hand, perceptions of workplace bullying can lead to changes in state anxiety without affecting trait anxiety. But, over time, repeated emotional fluctuations can also potentially form idiosyncratic states of anxiety. On the other hand, high trait anxiety cognitions are consistent with bullying situations, buffering the positive effects on state anxiety. However, according to the trait activation model, individuals with a predisposition to anxiety are more likely to develop recognition bias for danger signals and processing bias for emotional information (Van Bockstaele et al., 2014), thereby consuming additional control resources and reporting more negative perceptions. In the future, we should try to select a wide range of subjects and seek to generalize the research conclusions by combining research methods.

The second limitation is that, based on the appropriate theoretical basis and data analysis results, this paper recognizes a causal relationship between workplace bullying and job performance, but poor job performance is also potentially damaging to interpersonal relations (Hutchinson, 2012). Although empirical studies can be used to clarify the above causal relationship, individuals can conceal the negative emotions brought about by the rupture of the psychological contract and try to show their role performance in a way that is disproportionate to the objective crisis. It is therefore difficult to accurately test the behavior results of “passive resistance” in the cases of state anxiety.

One potential research direction in the future is to integrate previous theoretical models, which is the relationship between workplace bullying and state anxiety and the perspective of trait anxiety. Relevant literature on trait anxiety shows that trait anxiety can be reflected in the individual’s situation and existing experience (Spielberger, 1985), suggesting that the influence of workplace bullying on state anxiety is also likely to be moderated by interpersonal relationship positioning. The current research mainly focuses on the direct impact of workplace bullying on state anxiety, but individuals with low interpersonal sensitivity may not be affected too much by negative interpersonal treatment, and they may not show a strong increase in state anxiety even after workplace bullying.

A second area of future research is exploring other ways in which workplace bullying affects job performance. Although this paper has outlined the indirect influence path of workplace bullying on job performance through the mediation of state anxiety and the moderation of trait anxiety, there are other theoretical models to explain the influence of workplace bullying on work performance. For example, task interdependence can aggravate the negative impact of workplace bullying on job performance: when an individual’s task performance depends on the work input from other organizational members, the negative effect of workplace bullying on job performance will be more serious.

Finally, although the research emphasis of this paper focused on the individual difference variables that affect behavioral reaction after facing bullying, the current research has shown that a transformed situation perspective, such as promoting positive employee self-evaluation and self-view by encouraging feedback, and keeping a positive and optimistic state of mind (Wu, 2011), can also adjust negative perception of workplace bullying, prompting employees to do better in subsequent tasks. Thus, even in individuals with low trait anxiety, negative behavioral responses to workplace bullying may be neutralized through situational interventions.

## Conclusion

This study combined and reviewed results of workplace bullying, its negative influence, and the theoretical basis for staff behavior in the face of workplace bullying in choosing between “passive resistance” or “swallowing the insult.” It has proposed a moderated mediation model in order to better understand the mechanism of workplace bullying effects on job performance. The results of the data analysis reveal the mediating mechanism and moderating mechanism of those effects. Hence, the hypothesized model in this study may not only enhance the theoretical dialogue between workplace bullying, anxiety, ego depletion theory, and the theory of cognitive balance, but may also provide a relevant theoretical basis for promoting the development of practice.

## Data Availability Statement

All datasets generated for this study are included in the article/supplementary material.

## Ethics Statement

The study involving human participants were reviewed and approved by the ethical committee in School of Finance and Economics of Jiangsu University, Zhenjiang China. Written informed consent was not required in this study in accordance with the national legislation and institutional requirements. Also, the consent was inferred through the completion of the survey.

## Author Contributions

MW bring the idea. QH and MI completed all data collection. QH wrote the manuscript. MW, JF, and MI provided thoughtful feedback on the manuscript. All authors contributed to the design of the study and approved of this version of the manuscript.

## Conflict of Interest

The authors declare that the research was conducted in the absence of any commercial or financial relationships that could be construed as a potential conflict of interest.
